# An alternative high output tissue microarray technique

**DOI:** 10.1186/1746-1596-8-9

**Published:** 2013-01-21

**Authors:** Yuan Shi, Deming He, Yingyong Hou, Qin Hu, Chen Xu, Yalan Liu, Dongxian Jiang, Jieakesu Su, Haiying Zeng, Yunshan Tan

**Affiliations:** 1 Department of Pathology, Zhongshan Hospital, Fudan University, Fenglin Road #180, 200032, Shanghai, P.R. China

**Keywords:** Tissue rod, Tissue microarray, Technology, Sampling tool, High output

## Abstract

**Background:**

Tissue microarray (TMA) is a high throughput research tool, which has greatly facilitated and accelerated *in situ* tissue analyses. However, its productivity has been restricted due to the confined thickness of traditional donor block. Here, we introduce an improved high output TMA method that is applicable to a broader range of tissue samples.

**Methods:**

In this method, a 3.6 cm long and 2.7 cm wide recipient block with 88 square lattices (3 mm in width) was first prepared using several commercial instruments. A 2 mm wide and 6 mm long tissue rod was then prepared using a self-made blade-shaped knife from each paraffin embedded donor block of gastrointestinal stromal tumors. These rods were manually arrayed one by one into the corresponding lattices of the 60°C pre-softened recipient block with the guide of holes drilled with a steel needle. A 70-rod TMA was made to testify this method.

**Results:**

The prepared TMA had well defined array configurations, good tissue morphology and fully preserved proteins and DNA. A total of 500–1000 TMA sections could be easily obtained from a TMA block.

**Conclusion:**

This low-cost and time-saving method provides an alternative sampling tool for high output TMA.

**Virtual Slides:**

The virtual slide(s) for this article can be found here: http://www.diagnosticpathology.diagnomx.eu/vs/1979605867857990

## Introduction

In 1986, Battifora [1] proposed an ingenious idea for combining multiple tissue samples into a single “sausage” composite held together with a wrapper of intestinal casing. Wan [2] further modified the sausage technique by assembling the tissue cores manually extracted from parent tissue blocks without further deparaffinization. Several years after these pioneering and prototypical works of the modern tissue microarrays (TMAs), Kononen et al. [3] proposed and developed a novel high throughput TMA technique in 1998 to precisely re-embed up to 1,000 cores of donor tissue cylinders from a formalin-fixed paraffin-embedded “donor” block into a “recipient” paraffin block with the help of highly precise punching instruments. As a high throughput research tool, TMA, also known as tissue chip, is considered as a recent innovation in the field of pathology [4] and has greatly facilitated and accelerated *in situ* tissue analyses [5]. Sections from TMA can be stained for protein, DNA or RNA targets using *in situ* immunohistochemistry (IHC), fluorescence *in situ* hybridization (FISH) or mRNA *in situ* hybridization (RNA-ISH), respectively. The utility of TMA is generally found to be comparable to that of large sections [6]. TMA technique has dramatically changed traditional tissue sampling process of the above-mentioned *in situ* technologies, which are extremely tedious, time consuming, labor intensive and costly. For example, Schraml and coworkers [7] completed 3 FISH experiments on amplifications of three oncogenes in 3 × 397 tumors within a week.

TMA technology is extremely powerful, providing researchers the potential to derive extensive gene expression profiles invaluable particularly in the areas of tumor biology, clinical oncology and diagnostic test development [4,8-10]. In addition, TMA technology also plays an important role in educating pathologists about quality control in IHC and tissue banking [11].

Up to now, arrayers used for TMA are generally designed and supplied by several companies [12]. However, they are quite expensive. To achieve cheap TMA, Shebl et al. introduced an inexpensive mechanical pencil tip method for small paraffin tissue microarrays [13]. Although up to 1,000 different tissues can be analyzed in one TMA block, all TMAs are restricted by the thickness of paraffin embedded block because they are cylindrical cores obtained by punching the donor block. As a result, only about 100–300 sections are available from one TMA block [14]. In this study, we describe an alternative method using various tools including several in-house instruments to obtain rods of tissues from donor blocks and to create the recipient block with 88 small lattices. The rods of tissues were vertically embedded in the recipient block, which is fundamentally different from previous methods and could greatly improve the output of sections in one TMA block. Thus, it could be used as an alternative TMA approach for appropriate cases.

### Methodology

#### Selection of appropriate paraffin blocks with gastrointestinal stromal tumors (GISTs)

70 cases of GISTs were retrospectively collected from the department of pathology, Zhongshan Hospital, Fudan University. Prior written informed consent was signed by all patients and the study protocol was approved by the Ethics Committee Board at Zhongshan Hospital, Fudan University.

### Preparation of tissue “rod”

**Figure 1:**
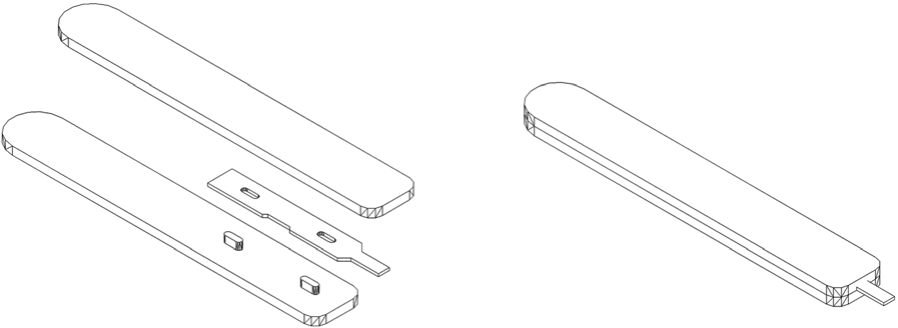
The picture of the tissue microarray sampling tool: a self-made blade-shaped knife transformed from a disposable microtome knife.

**Figure 2:**
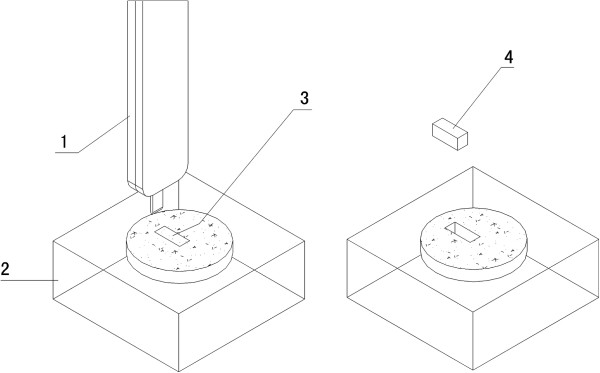
**A schematic presentation of the sampling procedure.** The interested area (3) was firstly identified on a donor tissue block (2), and then a rod of tissue (2 mm wide and 6 mm long) (4) was taken from the donor tissue block by sampling tool (1).

Tissue rods were prepared using a unique tissue microarray sampling tool (Chinese patent number: ZL201120336315.5), a blade-shaped knife self-made from disposable microtome knife (Figure 1). After identified on a donor tissue block using a hemotoxylin and eosin (H&E)-stained slide as a useful guide and marked with a marker pen, the region of interest (2 mm wide and 6 mm long) was extracted vertically down to the marked line from the donor tissue block (Figure 2) and placed into a 96 well culture plate in the position according to a chart prepared for recalling all the information about the tissue rods in the well.

### Preparation of recipient block and plantation of the donor tissue rods into the recipient block

**Figure 3:**
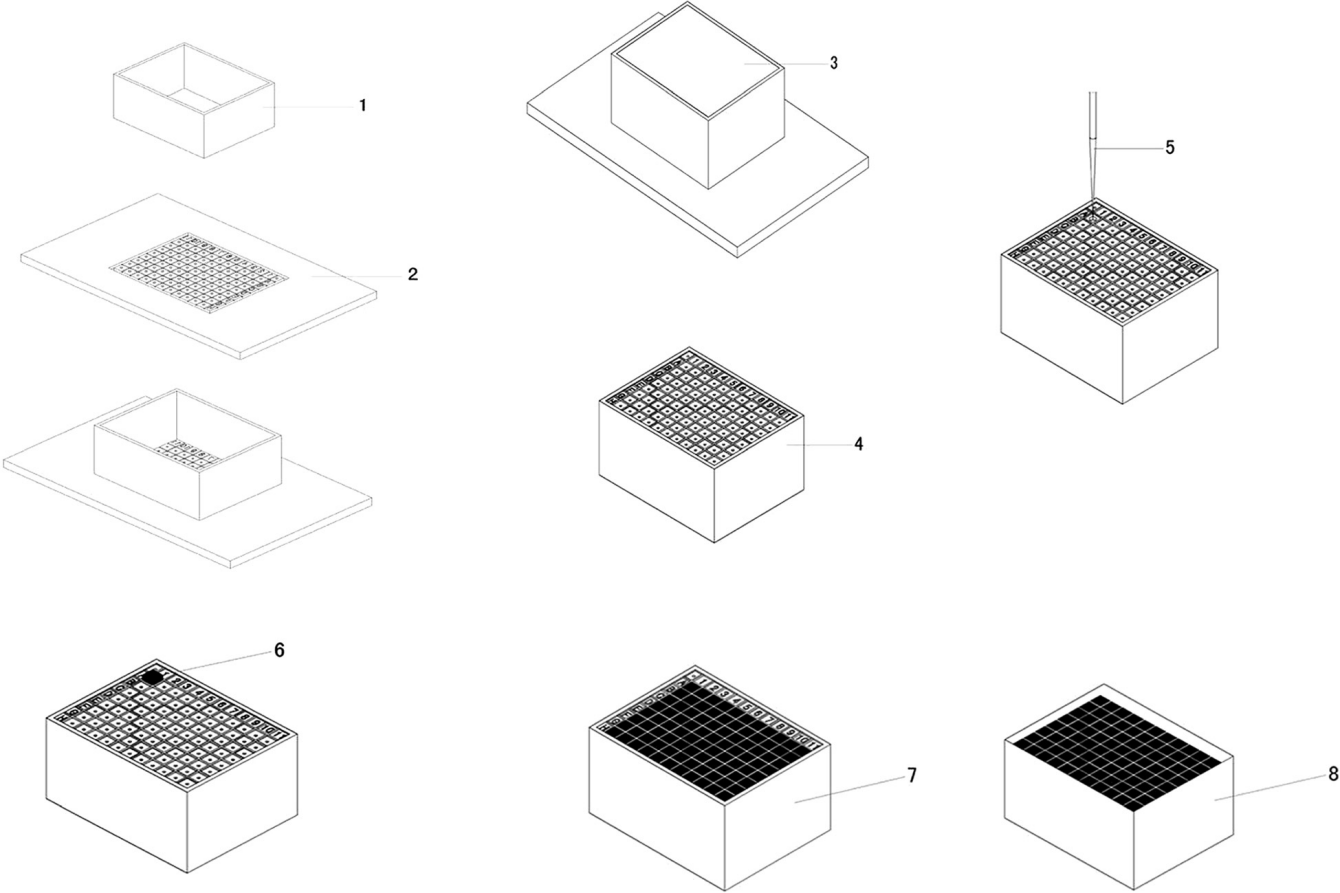
**TMA preparation procedure.** The plastic board (**2**) was placed under a topless and bottomless transparent box (**1**) and melting paraffin was poured into the box to cover the whole area (**3**) and cooled at room temperature till the plastic board could be separated from the transparent box (**4**). A hole was made by a guiding needle (**5**) on the block after the paraffin was softened at 60°C for 5 min. Tissue rods (**6**) were planted into the hole on the softened block one by one orderly until all the desired tissue rods were planted (**7**). After aggregating and cooling down, the TMA (**8**) was separated from the transparent box.

The steps of preparation and plantation of tissue rods were illustrated in (Figure 3). The checkerboard recipient block was a plastic board (3.6 cm in length and 2.7 cm in width) with 88 square lattices (3 mm in width). It was prepared using several simple instruments. The board was etched by laser to create 12 rows and 9 columns and marked as 1 to 11 in mirror phase in row except the first lattice and from A to H in mirror phase in column except the first lattice. After the plastic board was placed in a topless and bottomless transparent box, melting paraffin was poured into the box to cover the whole area and cooled at room temperature until the plastic board could be separated from the transparent box. The recipient block together with the transparent box was incubated at 60°C for 5 minutes prior to planting the rods of tissues to maintain the paraffin in a soft, but not melting status, and then placed on a flat board. Next, a hole was made at the starting spot A using a steel needle and the starting rod prepared by eosin staining was inserted into the drilled hole. Donor tissues stored in a 96 well culture plate were then manually planted into the recipient block one by one according to the corresponding location indicated by letters and numbers. The recipient block might be softened again in an incubator if hardened during the course of planting. The recipient block could firmly hold the rods of tissues without slanting the rods since the paraffin would be pushed to the former hole due to the restriction of the transparent box when drilling the next hole. The course of planting could be stopped in any spot until all the desired tissue rods were planted. The planting surface was aggregated on the aggregation instrument, which was important to ensure no bubbles appeared during the preparation. Then the recipient block with the transparent box was placed at 4°C for 10 min until the paraffin could be easily separated from the transparent box. Aftermath, the TMA recipient block was taken out and sectioned on a routine microtome machine. Slides in interval of 100 were chosen for H&E staining.

### Immunohistochemistry (IHC)

For the IHC, all incubations were performed at room temperature and washes were performed with TBST. Tissues sectioned at 5 μm on slides were dewaxed and rehydrated. Antigen retrieval was performed in a pressure cooker at 110°C for 5 min in a retrieval buffer (S2367, DAKO). Endogenous peroxidase activity was blocked with 3% hydrogen peroxide (S2023, DAKO). Sections were then incubated with CD117 antibody (DAKO) for 30 min and the signals were visualized with diaminobenzidine (K3468, DAKO) for 10 min.

### Analysis of *KIT* and *PDGFRA* Sequences

First, five consecutive sections were prepared and the same chip on the consecutive sections were collected for genetic test. The sequence analysis of *KIT* (exons 9, 11, 13, and 17) and *PDGFRA* (exons 12 and 18) was performed according to the protocols reported previously [15-17]. Primer sequences are available upon request. In short, genomic DNA was isolated from paraffin-embedded tissue samples using a standard phenol/chloroform organic extraction protocol. Sequencing reactions were conducted in both forward and reverse directions. The results were compared with the sequences of human *KIT* (NM_001093772) and *PDGFRA* (NM_006206) genes in National Center for Biotechnology Information.

## Results

### Section and H&E staining

**Figure 4:**
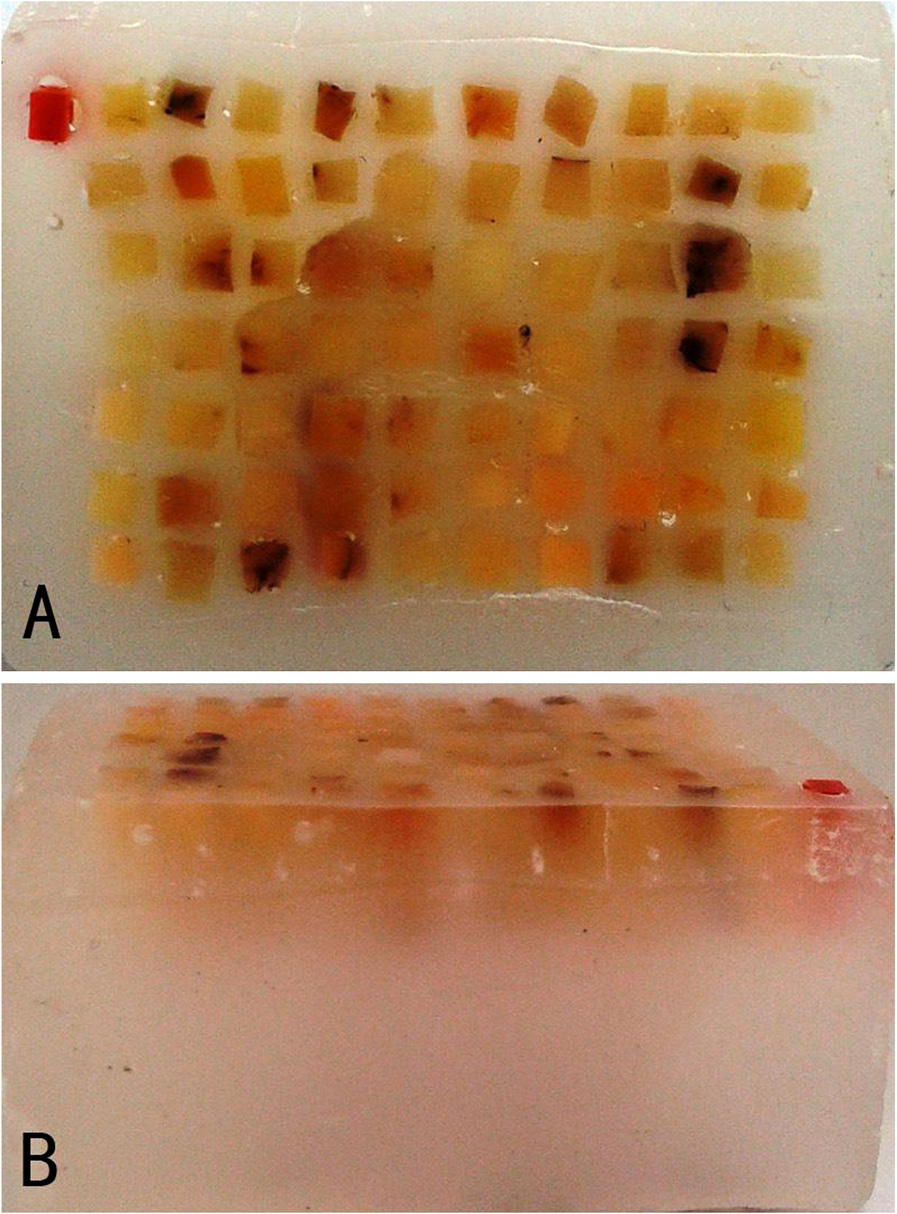
**Images of the prepared TMA. A**) Front view of a TMA of 3.6 cm long and 2.7 cm wide **B**) Lateral view of a TMA showing the tissue depth is vaguely visible.

**Figure 5:**
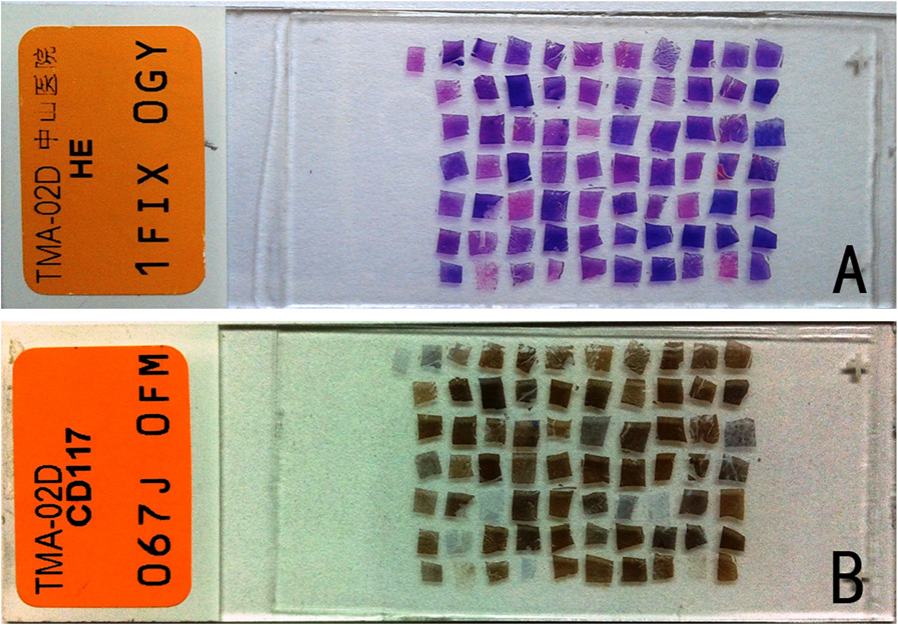
**Histological results of TMA samples. A**) H&E staining of TMA samples, showing a total of 70 spots in the section; **B**) IHC of TMA sections using CD117 antibody.

A 70-rod TMA (Figure 4A, 4B) was prepared as described above. Up to 1000 consecutive sections of 5 μm could be easily prepared by a trained histotechnologist. The prepared TMA could be sectioned repeatedly till the thickness was satisfied. These sections were adhered to a special slide and stained with H&E (Figure 5A). No missing spot was found after 200, 400, 800 and 1000 sections. The getting rate of the desired area was 100% in these sections. Morphology of all spots in the first section and the last one was consistent with that in the original slides.

### IHC on the TMA section

**Figure 6:**
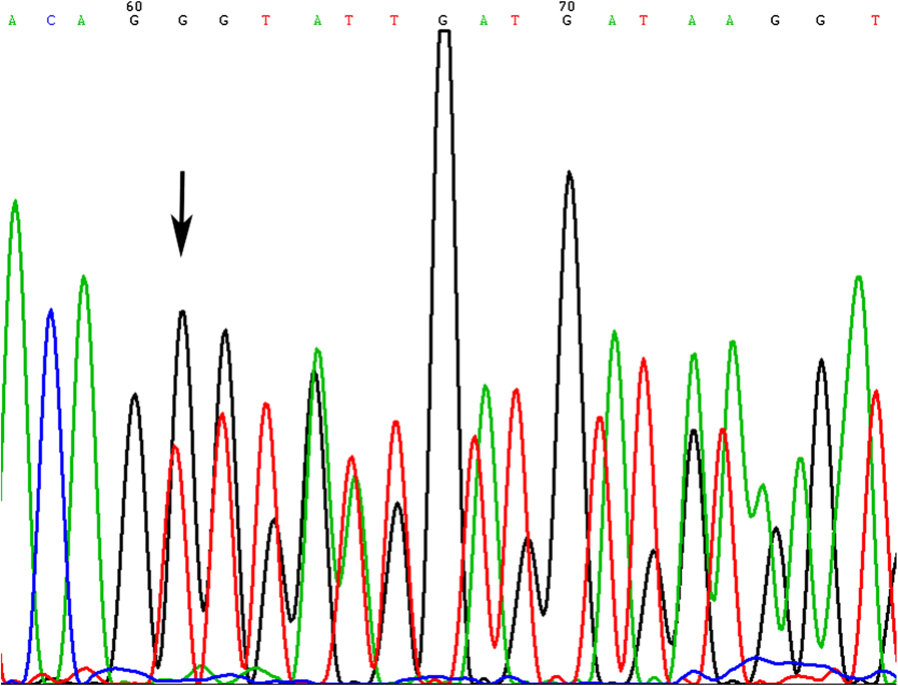
**Sequence of *KIT *gene at codons 557 and 558, showing the deletion of a TGGAAG in its exon 11.**

Among the 70 tumor tissues, 64 were positive (Figure 5B) and 6 were negative for CD117. All IHC results of TMA were consistent with those of their corresponding original slides.

### Analysis of *KIT* and *PDGFRA* sequences

The sequences of the analyzed genes obtained from TMA were consistent with those obtained from their corresponding original slides. Figure 6 shows TGGAAG deletion of the codons 557 and 558 in exon 11 of *KIT* gene.

## Discussion

### Development of the high throughput TMA

After 20 years of development, TMA technology has been well established in terms of both methodology and instruments [18]. In recent years, high-density TMA has become a standard laboratory tool for identifying and validating diagnostic and prognostic biomarkers for a variety of diseases, such as breast cancer, prostate cancer [9,19] and lung cancer. Application of 2 mm punchers allows construction of tissue arrays with about 50–100 specimens [14], while application of 0.6 mm punchers allows construction of a typical array of 300–500 specimens with a spacing of 0.8 mm between sample centers per block in regular tissue cassettes. A recipient block could accommodate up to 1,000 samples since the core of diameter can be as small as 0.43 mm [20]. Removing small diameter cores of tissue means that the tissue in the main block is preserved and does not significantly damage the structure of the tumor in the donor block, which can be used for further conventional sectioning should this become necessary. However, there are still several shortcomings in the conventional TMA block.

### Some shortcomings of the high throughput TMA

Firstly, tissues are embedded horizontally on the surface of a paraffin wax block with the thickness of tissue often less than 2–3 mm after being sectioned for H&E staining. If TMA is sectioned carefully by an experienced technician, at least 200 sections would be obtainable. Prior use of the donor block for immunohistochemistry as well as gene analysis in special cases may reduce this number, as the cores obtained will be shorter. Therefore, the productivity from one conventional TMA block is limited. Thus, the sections are often obtained at one time, otherwise at least ten sections would be lost because of trimming.

Secondly, tumor within the donor block is a three-dimensional structure, and so is the core. This means that the composition of any individual core may change as the sample is sectioned. A core may contain tumor cells at one end but only stroma at the other. If arrays are not well constructed or some cores are shorter than others, some cores will “sectioned out” before others. Previous investigators have reported a 10–30% loss of tissue when constructing conventional TMA from a large number of cores using manual or automated methods [7,21-23].

### Invention of a new high output TMA technique

The current new method could improve the output of TMA. As demonstrated, it is possible to construct a TMA from strip-shaped tissue rods taken from tumor abundant area and produce up to 1000 sections using appropriate simple tools. This method involves repositioning the rods in a vertical orientation, which enables the production of approximately 1000 sections from each rod of tissue to be used for H&E and IHC staining. Such sections could also, in principle, be used for other molecular procedures such as FISH. The IHC staining and DNA sequencing results showed that the TMA tissue maintained its antigens and DNA after the rods of tissues being planted in the recipient block.

Jhavar has described a method for preparing TMA from core biopsy tissues by sectioning small part of the tissues out of the prostate needle biopsy donor block and re-embedding the sections vertically in a new block [19]. Miettinen has also developed a simple method for generating multi-tissue blocks without special equipment [24]. However, these procedures are unsuitable for long rods of tissues. The rods of tissues could easily become slanted and are very difficult to orientate when re-embedded. Although our method is similar to theirs, it is more feasible for long rod of tissue. It took our 2 years to improve the method and ensure the rods could stand vertically in a TMA.

The present method of re-embedding tissue vertically is rare in TMA construction. A similar “checkerboard method” has been proposed by Battifora [1,25] to produce tissue arrays of a multi-tumor tissue block (MTB). In this checkerboard method, multiple (up to 100) chunks of formalin-fixed de-paraffinized or fresh normal or tumor tissues were reset first in agar and then in paraffin wax in a checkerboard pattern, and more than 1,000 sections with thickness of 5 μm could be obtained from one sausage type “MTB” block or checkerboard type “MTB” block. In our method, tissue rods were planted corresponding to the original samples rather than re-embedded samples in the chips.

### Advantages and disadvantages of our high output TMA technique

The advantages of our current approach are summarized as follows: a) the procedure and facility are simple, omitting hole-punching step, and no special, expensive instrument is required. With only a little practice, an operator could successfully select the needed rods from a “library” of tissues and plant them into a recipient paraffin block, b) the rods are almost immobile because they are long enough to stand stably, and c) the most improvement of the method is very high TMA output.

There are several practical limitations in vertically TMA construction. For example, pre-malignant lesions such as carcinoma *in situ* are not big enough for making high output TMA. On the other hand, carcinomas with large stromal component (such as pancreatic ductal adenocarcinoma) or large mucinous component (such as colorectal adenocarcinoma) contain a small proportion of viable cells on surface area; therefore they are not suitable for making high output TMA.

## Conclusion

Compared with punching cylindrical core-based TMA method, the high output TMA technology developed in this study appears to be relatively easy, time-saving and cost-efficient. Therefore, this method is more feasible for general research groups to prepare high output TMA slides from appropriate tissue samples.

## Competing interests

The authors declare that they have no competing interests. The authors don’t have any financial interests with any of the commercial products mentioned in this article.

The authors declare no conflict of interest to disclose.

## Authors’ contributions

This paper was carried out in collaboration between all authors. YS, YH and YT defined the research idea and participated in the design and helped draft the manuscript. DH, QH, CX, YL and DJ read the whole section and TMA slides. JS, HZ participated in the design and helped make the TMA sections. All authors read and approved the final manuscript.
